# A Sensor Fusion Approach to the Estimation of Instantaneous Velocity Using Single Wearable Sensor During Sprint

**DOI:** 10.3389/fbioe.2020.00838

**Published:** 2020-08-18

**Authors:** Salil Apte, Frederic Meyer, Vincent Gremeaux, Farzin Dadashi, Kamiar Aminian

**Affiliations:** ^1^Laboratory of Movement Analysis and Measurement, École Polytechnique Fédérale de Lausanne, Lausanne, Switzerland; ^2^Institute of Sport Sciences, University of Lausanne, Lausanne, Switzerland; ^3^Sport Medicine Unit, Division of Physical Medicine and Rehabilitation, Swiss Olympic Medical Center, Lausanne University Hospital, Lausanne, Switzerland; ^4^Gait Up S.A., Lausanne, Switzerland

**Keywords:** sensor fusion, sprinting, functional capacity test, wearable GNSS-IMU sensor, validation study, velocity profile, athlete monitoring

## Abstract

Power-Force-Velocity profile obtained during a sprint test is crucial for designing personalized training and evaluating injury risks. Estimation of instantaneous velocity is requisite for developing these profiles and the predominant method for this estimation assumes it to have a first order exponential behavior. While this method remains appropriate for maximal sprints, the sprint velocity profile may not always show a first-order exponential behavior. Alternately, velocity profile has been estimated using inertial sensors, with a speed radar, or a smartphone application. Existing methods either relied on the exponential behavior or timing gates for drift removal, or estimated only the mean velocity. Thus, there is a need for a more flexible and appropriate approach, allowing for instantaneous velocity estimation during sprint tests. The proposed method aims to solve this problem using a sensor fusion approach, by combining the signals from wearable Global Navigation Satellite System (GNSS) and inertial measurement unit (IMU) sensors. We collected data from nine elite sprinters, equipped with a wearable GNSS-IMU sensor, who ran two trials each of 60 and 30/40 m sprints. We developed an algorithm using a gradient descent-based orientation filter, which simplified our model to a linear one-dimensional model, thus allowing us to use a simple Kalman filter (KF) for velocity estimation. We used two cascaded KFs, to segment the sprint data precisely, and to estimate the velocity and the sprint duration, respectively. We validated the estimated velocity and duration with speed radar and photocell data as reference. The median RMS error for the estimated velocity ranged from 6 to 8%, while that for the estimated sprint duration lied between 0.1 and −6.0%. The Bland–Altman plot showed close agreement between the estimated and the reference values of maximum velocity. Examination of fitting errors indicated a second order exponential behavior for the sprint velocity profile, unlike the first order behavior previously suggested in literature. The proposed sensor-fusion algorithm is valid to compute an accurate velocity profile with respect to the radar; it can compensate for and improve upon the accuracy of the individual IMU and GNSS velocities. This method thus enables the use of wearable sensors in the analysis of sprint test.

## Introduction

Sprinting not only represents the peak of human speed but also forms the basis of performance in a variety of sports. The capacity to generate maximal force and power in the direction of running is a decisive factor behind an athlete’s performance in sports such as athletics, soccer, hockey, rugby, etc. (Cronin and Hansen, 2005). To ascertain this capacity, sprint tests with a distance varying from 20 to 60 m are typically utilized. A wealth of research into sprint mechanics (Morin et al., 2012; Buchheit et al., 2014; Cross et al., 2015; Rabita et al., 2015; Haugen and Martin, 2016) has shown that parameters such as maximum power produced by the sprinter, maximum horizontal force, horizontal velocity at zero acceleration, maximum theoretical horizontal force (*f*_0_), maximum theoretical horizontal power (*p*_*max*_), maximum theoretical horizontal velocity (*v*_0_) etc., along with the horizontal force-velocity (F-V) and horizontal power-velocity (P-V) profiles can be crucial for designing personalized training programs, evaluating injury risks, and athlete readiness to resume high intensity training and return to competition after injury (Morin and Samozino, 2016). These parameters and the force-power-velocity profiles can be ascertained using the velocity profile during sprint. An accurate estimation of the in-field sprinting velocity can thus be immensely helpful to improve the performance of athletes in a multitude of sports.

The prominent model of estimating instantaneous sprint velocity (*v*_mdl_(*t*)) is based on the use of a Doppler radar to measure the maximum velocity in combination with the Eq. 1 (Furusawa et al., 1927; Samozino et al., 2016):

(1) $$v_{m\,d\,l}\,\left(t\right)=v_{m\,a\,x}\,\left(1-e^{\left\{-\frac{t}{\tau }\right\}}\right)$$

where *v*_*max*_ is the maximum horizontal velocity during the sprint and τ is a constant, estimated using ensemble experimental data. The obtained velocity profile (*v*_*mdl*_(*t*)) is differentiated to obtain horizontal acceleration, and subsequently the F-V and P-V profiles. While this method provides ease of use, it is only valid when the athletes can approach or attain *v*_*max*_. However, the sprinters may not achieve *v*_*max*_ over short distances such as 30 m or they may not be able to maintain *v*_*max*_ over longer distances such as 60–100 m, especially during training sessions, and thus the sprint velocity profile for all athletes may not necessarily show a first-order exponential behavior. Sprint velocity has also been estimated with a recently developed application (Stanton et al., 2016) for a smartphone; wherein the in-built camera tracks and records the motion. Based on the distance entered manually, the application calculates the total sprint time and subsequently the mean velocity. Thus, this application cannot estimate instantaneous velocity and the measurable sprint distance might be limited by the field-of-view of the camera.

While wearable inertial sensors have shown promising results in the assessment of temporal gait parameters in running and sprinting (Leitch et al., 2011; Bergamini et al., 2012; Norris et al., 2014; Falbriard et al., 2018; Macadam et al., 2019), their use for analysis of instantaneous sprint velocity and other sprint mechanics has been rather rare. Recently, a magnetic and inertial measurement unit (MIMU) based algorithm (Setuain et al., 2018) has been developed to assess sprint mechanics with various parameters such as maximal velocity, maximal horizontal force and power, velocity at zero horizontal force, etc., for 20 m sprints. Though this work allows the measurement of sprint mechanics using a single MIMU mounted on the trunk, the algorithm relies on the use of split times from photocells at specific distances to remove the accumulated drift in the velocity. Other works on velocity estimation using a trunk-based MIMU (Gurchiek et al., 2018, 2019), utilized Eq. 1 for drift removal and used machine learning to estimate the parameters *v*_*max*_ and τ, respectively. Nevertheless, as explained earlier, Eq. 1 may not hold true over different sprint distances and sub-maximal efforts. Finally, Global Navigation Satellite System (GNSS) with wearable receiver provides another avenue of running velocity measurement in field and has been used to assess training and match performance in sports like soccer and rugby (Cummins et al., 2013). However, the ground velocity signal from GNSS is not responsive enough to measure the velocity during sprint (Nagahara et al., 2017) and can lead to an underestimation of the sprint velocity. This issue is even more exacerbated among elite athletes, who produce a high magnitude of horizontal acceleration and for whom, the timing difference can be critical (Morin and Samozino, 2016).

A Kalman filter based sensor fusion approach to combine GNSS and MIMU signals can overcome their respective limitations of responsiveness and drift-induced errors, as demonstrated successfully in sports applications such as skiing (Waegli et al., 2007; Brodie et al., 2008; Zihajehzadeh et al., 2015) and running (Tan et al., 2008). However, the works on skiing utilized magnetometers and focused on estimating and validating the skier’s trajectory and not the velocity, whereas the running movement did not present the challenge of high starting acceleration encountered in sprinting. Use of sprinting as a functional capacity test also imposes an important constraint in terms of usability for in-field implementation, thus limiting the number of wearables that can be utilized.

To address the problem of estimating instantaneous velocity in sprinting over a range of distances, we introduce a new approach based on using a gradient descent algorithm as an orientation filter (Madgwick et al., 2011), in combination with cascaded simple Kalman filters used for precise data segmentation and velocity estimation, respectively. The orientation filter utilizes the IMU data to convert the acceleration signals from the sensor frame to the global frame, which is then given as input to the first Kalman filter for estimating the precise sprint duration. This duration is used to segment the sensor data, which is then provided to the second Kalman filter, which fuses the GNSS signal and IMU acceleration to estimate the instantaneous velocity. To test this approach, we used the instantaneous velocity obtained from a Doppler effect-based radar for validating the estimated velocity and sprint timings acquired from a photocell for comparing the sprint duration.

## Materials and Equipment

**FIGURE 1:**
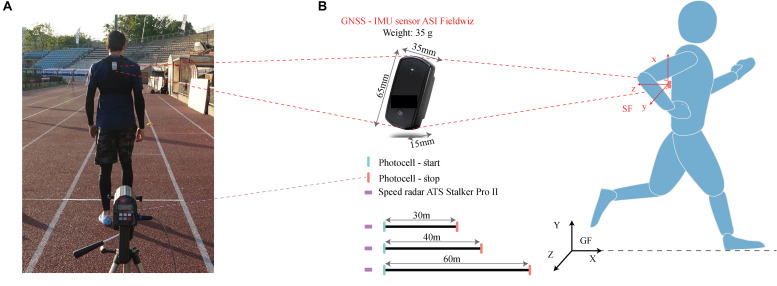
Sensor setup and measurement protocol, **(A)** Snapshot of a sprinter wearing the *Fieldwiz* sensor with the speed radar in the foreground **(B)** Specifications of the Fieldwiz sensor and the measurement protocol, wherein the sprinters ran two trials each of 60 m and 30 or 40 m distances with the speed radar as the velocity reference. Photocells were positioned at the start/end to record the sprint duration. GF and SF represent the global and sensor frames.

We conducted measurements with nine healthy elite-level sprinters, four (3 male, 1 female, 60 m sprint time 7.49 ± 0.35 s) at the Aix-les-Bains Athletics club and five (4 male, 1 female, 60 m sprint time 7.65 ± 0.67 s) from the Lausanne Athletics club, respectively. Ethical approval for the study was obtained from the university human research ethics committee (HREC 039-2018) and prior written consent was obtained from all the participants. The Aix-les-Bains cohort performed 2 × 40 m and 2 × 60 m sprints, while the Lausanne one performed 2 × 30 m and 2 × 60 m sprints. These distances are typically used in sprint tests and for training sprinters. For both measurements, participants were wearing a vest equipped with the GNSS-IMU sensor (Fieldwiz, ASI, CH) on the upper back (Figure 1); GNSS here represents the GNSS and IMU the inertial measurement unit. Apart from the vest, the sprinters dressed as they would for a regular training session.

This GNSS-IMU wearable sensor was chosen because it is already used in soccer training for performance and training monitoring (Clemente et al., 2018). This sensor, with a sampling frequency of 200 Hz for the IMU and 10 Hz for the GNSS unit, was used in the “airborne <4 g” configuration of the in-built *u-blox* GNSS module. A speed radar (ATS Pro II, Stalker Sport, United States) with a sampling frequency of 50 Hz, selected on the basis of Haugen and Martin (2016), was positioned directly behind the starting point (Figure 1) of the sprinter. Data from the radar was used in the measurements as a reference value for velocity. Photocells (Witty, Microgate corp, Italy) from the respective athletics clubs were used at the start and the end as reference value for the duration of the sprints.

## Methods

### Velocity and Duration Estimation Algorithm

**FIGURE 2:**
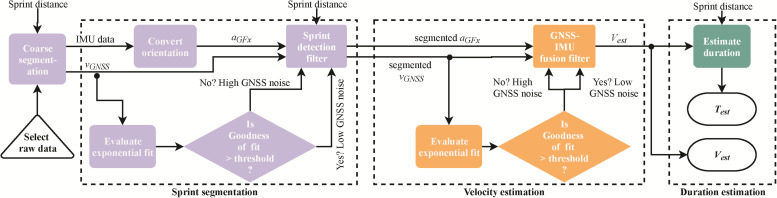
Flowchart for the sprint velocity estimation algorithm. The “coarse segmentation” block is manual and creates a window to select the approximate starting point of the relevant sprint, while remaining algorithm is automated. The “Sprint detection” and “GNSS-IMU fusion” filters are simple Kalman filters. *a*_**GFx**_ denotes the horizontal acceleration in the global frame, *v*_*GNSS*_ the ground velocity from the GNSS sensor, while *v*_*est*_ and *T*_*est*_ represent the estimated velocity and sprint duration, respectively.

**FIGURE A1:**
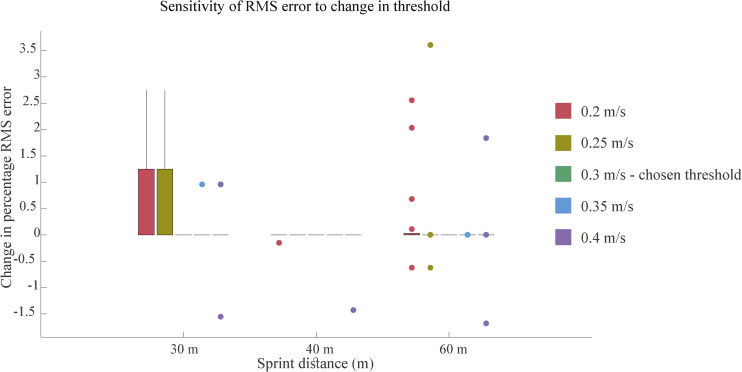
Change in the percentage of RMS error from its value at the chosen threshold of 0.3 m/s. The RMS error is slightly sensitive to the threshold for the 30 m sprint, especially when the threshold is below 0.3 m/s. For other distances, the error is almost insensitive to the threshold.

The flowchart for the algorithm is shown in Figure 2; the algorithm includes three phases: (i) sprint segmentation (ii) velocity estimation, and (iii) sprint duration estimation. Sprint segmentation aims to detect the period for each specific sprint. First, the data recorded on the GNSS-IMU sensor is segmented by manually selecting an approximate starting sample for the relevant sprint. Following this, the algorithm is designed to choose a precise *starting time* (*t*_*s*_) by selecting an appropriate threshold (0.3 m/s) on the velocity obtained from the GNSS sensor. A sensitivity analysis (Appendix Figure A1) was conducted to see the impact of this threshold on the velocity estimation error. Using gravity and the IMU data during the static period at the start of sprint, the initial orientation is estimated along *X* and *Y* direction, wherein the direction of sprinter progression is assumed to be the global *X*-axis and *Y* is the vertical axis. The changes from this initial orientation are estimated using the gyroscope data and corrected with the accelerometer data using a gradient-descent based optimization method (Madgwick et al., 2011). Thus, the *X*-axis here is not truly a global axis and it is defined anew for every sprint. The changes in orientation are represented by quaternions *q*, which are used to convert the acceleration signals from the segmented data from the sensor frame (SF) to the global frame (GF) *X*–*Y*–*Z* using Eq. 2:

(2) $$a_{G\,F}=q⊗\left[0\;a_{S\,F}\right]⊗q^{*}$$

Where *q* represents the quaternions transforming the sensor frame (SF) to the global frame (GF) and *q*^∗^ their transpose. These quaternions are estimated by fusing accelerometer and gyroscope data using a gradient descent algorithm (Madgwick et al., 2011); *a*_*SF*_ is the acceleration in the sensor frame, and *a*_*GF*_ is the acceleration in the global frame *X*–*Y*–*Z* with positive *X*-axis representing the direction of sprinting.

The acceleration along the positive *X*-axis of the global frame (*a*_*GFx*_) is provided as an input to the Sprint detection filter (linear Kalman filter) in combination with the ground velocity (*v*_*GNSS*_) from the GNSS sensor. The main assumption here is that the sprinters run along a straight line (within sagittal plane), thus the acceleration (*a*_*GFx*_) can be assumed to represent acceleration along the direction of running and the dynamical model of the system can be assumed to be constant. This assumption is also used during the measurements with a speed radar; in our case, it simplified the system to a linear model and allowed the use of a simple Kalman filter, which is the optimal estimator for a linear system (Burl, 1998). This filter has the following prediction and update steps:

*Prediction*:

(3.1) $$v_{e\,s\,t}\,\left(n|n-1\right)=\left[1\right]\,v_{e\,s\,t}\,\left(n-1\right)+\left[\Delta \,t\right]\,a_{G\,F\,x}\,\left(n-1\right)+\mu$$

Update:

$$v_{e\,s\,t}\,\left(n|n\right)=v_{e\,s\,t}\,\left(n|n-1\right)$$

(3.2) $$+K\,\left(n\right)\,\left(v_{G\,N\,S\,S}\,\left(n\right)-v_{e\,s\,t}\,\left(n|n-1\right)\right)$$

*Kalman gain*:

(4) $$K\,\left(n\right)=p\,\left(n|n-1\right)\,{\left(p\,\left(n|n-1\right)+\eta \right)}^{-1}$$

Where *v*_*est*_ is the estimated horizontal velocity, *a*_*GFx*_(*n*) is the horizontal acceleration in global frame, Δ*t* is the sampling time, μ is the process (accelerometer) noise, *v*_*GNSS*_(*n*) is the velocity measured by the GNSS sensor, *K*(*n*) is the Kalman gain, *p*(*n*) is the estimation uncertainty, and η is the measurement (GNSS) noise. Since *a*_*GFx*_ has a sampling frequency of 200 Hz, *v*_*GNSS*_ is upsampled from 50 to 200 Hz by “zero padding.” If the velocity from *v*_*GNSS*_ is non-zero, the update sequence is initiated, otherwise the prediction model continues to run without update.

The magnitudes of η and μ were set to 0.01 and 0.4, respectively, obtained via manual tuning of the filter. In order to refine the magnitude of η further, the rationale of the exponential behavior of sprint velocity (Samozino et al., 2016) is utilized. By subtracting both sides of Eq. 1 from *v*_*max*_, we get:

(5) $$v_{m\,a\,x}-v_{H}\,\left(t\right)=v_{m\,a\,x}\,\left(e^{\left\{-\frac{t}{\tau }\right\}}\right)$$

Based on this equation, *v*_*GNSS*_ is subtracted from the maximum velocity and an exponential curve was fitted to it and if fit is good (*R*^2^ > 0.91), the value of η_*k*_ is unchanged from 0.01. In case of a bad fit, this value is increased by an order of magnitude to 0.1. The velocity (*v*_*est*_) obtained from this Kalman filter is integrated from the starting time (*t*_*s*_) to obtain the distance profile, which is subsequently compared to the actual sprint distance and used to estimate the ending time *t*_*e*_ and segment sprint period (*t*_*d*_ = *t*_*e*_−*t*_*s*_) precisely.

In the second phase, a more accurate exponential fitting is made using a more refined sprint period (*t*_*d*_) obtained in the first phase. Precisely segmented *v*_*GNSS*_ and *a*_*GFx*_ are provided as inputs to the GNSS-IMU fusion filter, which is also a simple Kalman filter, with the same process and measurement models as the first filter. This filter is used to update the final sprint velocity (*v*_*est*_) precisely by considering the sprint period and the fine-tuning of GNSS noise. In the final step, *v*_*est*_ is integrated to obtain the displacement-time profile and the timestamp at the relevant sprint distance is computed. The starting time (*t*_*s*_) of the sprint is then subtracted from the value of this timestamp to obtain the sprint duration (*T*_est_).

### Estimation of Profiles – Velocity, Force, and Power

To estimate force-velocity and power-velocity profiles, the first step is to estimate the approximate velocity profile from *v*_*est*_ using the exponential fit (Samozino et al., 2016) presented in Eq. 1. While the maximum velocity during the sprint (*v*_*max*_)and the velocity at the end (*v*_*end*_) are the same in case of an ideal exponential velocity profile, this may not be the case with real-world velocity profiles. As a result, *v*_*max*_ and *v*_*end*_ tend to deviate from each other. To investigate which velocity profile leads to a better fit, the two first-order velocity profiles, based on *v*_*max*_(*v*_*mdl*_*max*,1_(*t*)) and *v*_*end*_(*v*_*mdl*_*end*,1_(*t*)), respectively, were compared to a second-order velocity profile, defined as:

(6) $$v_{m\,d\,l,2}\,\left(t\right)=a\,e^{\tau_{1}\,t}-a\,e^{\tau_{2}\,t}$$

Where τ_*1*_, τ_*2*_ and *a* were computed with the “*trust-region reflective*” algorithm, using the “*lsqcurvefit*” function native to Matlab application. Approximate velocity profile obtained from the best performing fitting method is differentiated to obtain the approximate horizontal acceleration *a*_*mdl*_(*t*), which in combination with the sprinter’s mass (*M*), led to the force profile:

(7) $$F_{m\,d\,l}\,\left(t\right)=M\,a_{m\,d\,l}\,\left(t\right)$$

Finally, we computed the power profile as a product this force profile and the velocity profile:

(8) $$P_{m\,d\,l}\,\left(t\right)=F_{m\,d\,l}\,\left(t\right)\,a_{m\,d\,l}\,\left(t\right)$$

### Validation Process

The velocity measured at 50 Hz by the radar (*v*_*R*_(*t*)) was used as reference for velocity validation. To match the sampling frequency of the reference signal, *v*_*est*_ was downsampled from 200 to 50 Hz by keeping the first sample and every fifth sample after the first, and *v*_*GNSS*_ was upsampled from 10 to 50 Hz using linear interpolation. An error vector (Eq. 9) between *v*_*est*_ and *v*_*R*_ was then computed for each trial. Following this, the RMS for each error vector were calculated. Finally, median and interquartile range (IQR) were computed from the RMS error value for each sprint distance to investigate the bias and precision. Similar procedure was applied to estimate error for *v*_*GNSS*_.

(9) $$\varepsilon_{v}\,\left(t\right)=\frac{v_{R}\,\left(t\right)-v_{e\,s\,t}\,\left(t\right)}{m\,a\,x\,\left(v_{R}\,\left(t\right)\right)}\times 100\%$$

In order to investigate the different fitting methods explained earlier, we calculated the error vectors (Eq. 10) of the fitted curves *v*_*mdl*_(*t*) [i.e., *v*_*mdl*_*max*,1_(*t*), *v*_*mdl*_*end*,1_(*t*) and *v*_*mdl*,2_(*t*)] with respect to *v*_*R*_, followed by calculating RMS, median, and IQR. Further, we also investigated the fitting performance qualitatively by observing the different fitted velocity profile curves. Similarly, the error for fitted curves with respect to *v*_*est*_ was calculated.

(10) $$\varepsilon_{f\,i\,t}\,\left(t\right)=v_{R}\,\left(t\right)-v_{f\,i\,t}\,\left(t\right)$$

The time recorded in the photocells (*T*_*Ref*_) was used as reference for validation of the estimated sprint duration (*T*_*est*_). Percentage error for the sprint duration was calculated by Eq. 11:

(11) $$\varepsilon_{t}=\frac{T_{R\,e\,f}-T_{e\,s\,t}}{T_{R\,e\,f}}\times 100\%$$

Similar process was carried out for the duration obtained from the radar (*T*_*rad*_), in order to compare the performance of the algorithm with that of the radar. Subsequently, the RMS, median, and IQR for these error values were calculated.

Lastly, the maximum velocity is an important metric according to earlier research on sprint mechanics (Morin et al., 2012) and thus, we opted to compare the value obtained from our method with that from the radar. Another reason to focus on the maximal speed was that the RMS error did not capture this parameter properly. The Bland–Altman plot (mean-difference) was used (Altman, 1990) for this purpose, along with the calculation of the Lin’s concordance correlation coefficient (ccc) at 95% confidence interval (Lawrence and Lin, 1989) as a measure of agreement between our method and the radar. A correlation coefficient value greater than 0.7 was considered “strong,” according to the ranges suggested in Hopkins et al. (2009) for sports science research. Bland–Altman plots were also utilized to compare the theoretical maximum theoretical velocity *v*_0_ (m/s), maximum theoretical horizontal force per unit mass *f*_0_ (N/kg), and maximum theoretical horizontal power *p*_*max*_ per unit mass (W/kg) values obtained from the *v*_*est*_(*t*) using the second-order exponential fit to those computed from the *v*_*R*_(*t*). The *p*_*max*_ values were obtained from the apex values of the P-V profile.

## Results

Data for the nine athletes (7 male, 2 female, 60 m sprint time 7.39 ± 0.37 s) was utilized in this research. Four athletes performed 2 × 40 m sprints and 2 × 60 m sprints, while remaining five athletes performed 2 × 30 m sprint and 2 × 60 m sprints. For one 60 m sprint and three 30 m sprints, a delay in triggering the reference radar system was noticed during data processing. Since the sprint start was not recorded for these sprints, their data was discarded from the final analysis. Thus, a total seven sprints were considered for 30 m distance, eight for 40 m, and 17 for 60 m. Out of these, data for two 40 m sprints was used for tuning the algorithm, while the data for all sprints was used for validation.

### Velocity Estimation

**FIGURE 3:**
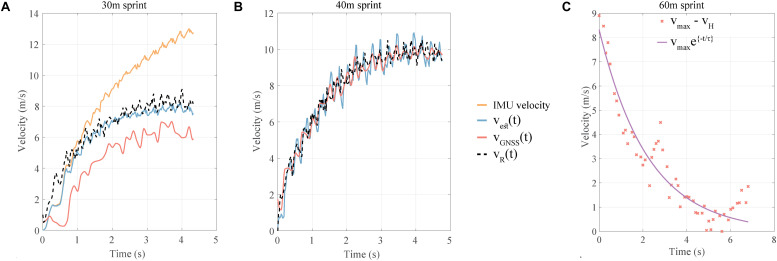
**(A)** Example of a specific case of 30 m sprint when *v*_*GNSS*_(*t*) was inaccurate while the estimated velocity is accurate. **(B)** Example of a specific case of 40 m sprint when *v*_*GNSS*_(*t*) was accurate and so was the estimated velocity. **(C)** Example of exponential fit (Eq. 5) used to adjust measurement (GNSS) noise for the Kalman filter. IMU velocity: velocity obtained by strapdown integration of IMU signals, *v*_*GNSS*_(*t*): GNSS velocity, *v*_*R*_(*t*): radar velocity, *v*_*est*_(*t*): estimated velocity by GNSS-IMU fusion.

Figure 3 illustrates one example each of situations where *v*_*GNSS*_ severely underestimated the actual *v*_*R*_ (Figure 3A) and when the *v*_*GNSS*_ approximately matches *v*_*R*_ (Figure 3B). In both cases, *v*_*est*_ matched *v*_*R*_ closely. Figure 3C, in turn, represents the intermediate ‘Evaluate exponential fit’ block of the algorithm (Figure 2 and Eq. 5), for adjusting the measurement noise parameter of the Kalman filter. For the case presented here, *v*_*GNSS*_(*t*) did not show an exponential behavior (*R*^2^ = 0.66) and so the measurement noise, (η = 0.1) was set higher than scenario when *v*_*GNSS*_(*t*) would have been exponential (*R*^2^ > 0.91) in nature. Apart from this one case of 30 m, *v*_*GNSS*_(*t*) did not show an exponential behavior in one of 40 m sprints.

#### Validity of Estimated Velocity

**FIGURE 4:**
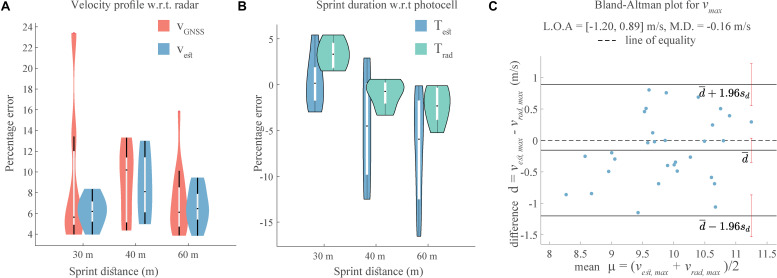
Validation of estimated velocity, **(A)** RMS error of the estimated velocity and GNSS velocity w.r.t. the radar speed. **(B)** RMS error of the predicted sprint duration from the proposed algorithm and the radar speed with the photocell duration as reference. **(C)** Bland–Altman plot for the maximum estimated velocity with the maximum radar speed as reference. Here, L.O.A. are the limits of agreement and M.D. is the mean difference.

The error results for *v*_*est*_ and *v*_*GNSS*_ are shown in Figure 4 and Table 1; *v*_*est*_ presents a similar error magnitude as *v*_*GNSS*_ for 40 and 60 m, while showing a lower error for the 30 m sprint. The median of RMS errors of the *v*_*est*_ ranged from 6.2 to 8.1% (Figure 4A and Table 1) for the three sprint distances and was lower or similar to that of the *v*_*GNSS*_. Furthermore, the IQR (Table 1) for the RMS errors for the *v*_*est*_ was lower than that of the *v*_*GNSS*_, especially for the 30 and 60 m sprint distances.

**TABLE 1:** Median (IQR) values of the RMS error for *v*_*GNSS*_,*v*_*est*_,*T*_*rad*_*andT*_*est*_ for all three sprint distances.

**Sprint distance, m**	**% error for *v*_*GNSS*_**	**% error for *v*_*est*_**	**% error for *T*_*rad*_**	**% error for *T*_*est*_**
30	5.6 (4.9 to 12.0)	6.2 (5.2 to 7.2)	3.3 (1.8 to 4.5)	0.1 (−1.7 to 1.9)
40	10.2 (5.1 to 11.4)	8.1 (6.1 to 11.4)	−0.8 (−2.0 to 0.2)	−4.5 (−9.8 to 0.1)
60	6.1 (4.7 to 8.5)	6.5 (5.4 to 7.9)	−2.1 (−3.4 to −0.2)	−6.3 (−12.8 to −2.4)

*RMS error was calculated on the basis of Eqs 9 and 11.*

**FIGURE 5:**
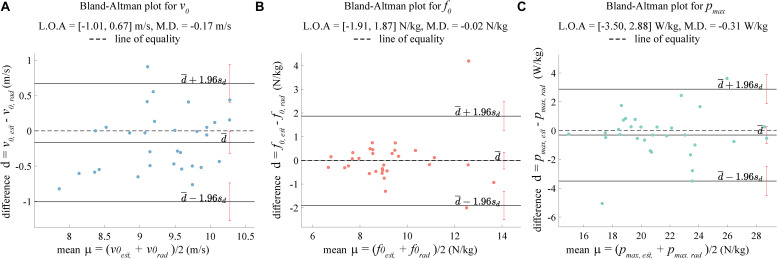
Bland–Altman plots with the values calculated from radar speed as reference, where L.O.A. are the limits of agreement and M.D. is the mean difference. The values here are obtained using the second-order exponential fit, **(A)** Maximum theoretical velocity *v*_0_ (m/s). **(B)** Maximum theoretical horizontal force per unit mass *f*_0_ (N/kg). **(C)** Maximum theoretical horizontal power *p*_*max*_ per unit mass (W/kg).

The median error for *T*_*est*_ ranged from 0.1 to −6.3% (Figure 4B), while that for *T*_*est*_ varied from 3.3% to −2.3%, thus both showed a similar range. The IQR (Table 1) for *T*_*rad*_ were lower as compared to *T*_*est*_ for 40 and 60 m sprints. For 30 m sprint, *T*_*est*_ had a lower median error, but a higher IQR than *T*_*rad*_. For the maximum velocity (*v*_*max*_), the Bland–Altman plot showed close agreement between the estimated and the reference magnitudes, with all the values lying between the two standard deviations and the Lin’s concordance correlation coefficient being 0.76 (*p* < 0.05). The estimated values, however, showed a slight negative bias of −0.16 m/s, although this was miniscule as compared to actual maximum velocities, which were around 10 m/s. For the *v*_0_, *f*_0_, and *p*_*max*_, the Bland–Altman plot (Figure 5A) showed close agreement between the estimated and reference values, with almost all values lying between the two standard deviations. *v*_0_ presented a bias of −0.17 m/s which is similar to that of *v*_*max*_, *f*_0_ showed almost zero bias, and the bias for *p*_*max*_ was −0.31 W/kg, which is substantially smaller than the actual *p*_*max*_ values, which range from 16 to 28 W/kg.

### Validity of Exponential Fitting

**FIGURE 6:**
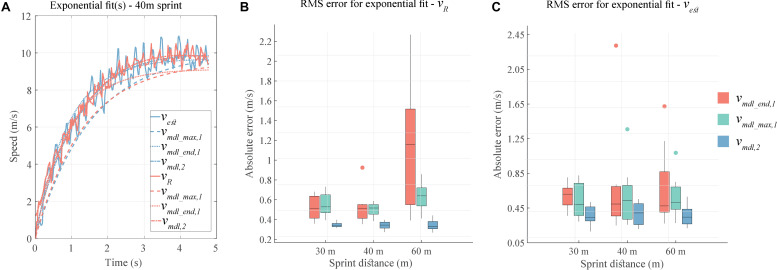
**(A)** Three methods for exponential fit. **(B)** RMS error for exponential fit(s) on radar speed (*v*_*R*_). **(C)** RMS error for exponential fit(s) on estimated velocity (*v*_*est*_).

A qualitative presentation of the different types of exponential fits can be seen in Figure 6A, for the first order (*v*_*mdl*_*max*,1_,*v*_*mdl*_*end*,1_) and second order (*v*_*mdl*,2_)exponential fits.

For both *v*_*est*_ and *v*_*R*_, the second order fit has the lowest RMS error (Figures 6B,C) and lower median and IQR than both first order fits (Table 2). *v*_*mdl_ end,1*_ fit has similar median error values as *v*_*mdl_ max,1*_ fit for 30 and 40 m sprints, while it has considerably higher median and IQR for the 60 m sprint (Table 2).

**TABLE 2:** Median (IQR) values for the RMS error in the three types of exponential fits, for all three sprint distances.

**Sprint dist., m**	***v*_*mdl_ max,1*_**		***v*_*mdl_ end,1*_**		***v*_*mdl,2*_**	
	**Fit on *v*_*R*_**	**Fit on *v*_*est*_**	**Fit on *v*_*R*_**	**Fit on *v*_*est*_**	**Fit on *v*_*R*_**	**Fit on *v*_*est*_**
30	0.53 (0.47 to 0.65)	0.49 (0.36 to 0.74)	0.51 (0.41 to 0.64)	0.61 (0.48 to 0.68)	0.34 (0.33 to 0.36)	0.34 (0.30 to 0.46)
40	0.52 (0.46 to 0.55)	0.53 (0.32 to 0.71)	0.51 (0.41 to 0.55)	0.50 (0.36 to 0.70)	0.34 (0.31 to 0.37)	0.40 (0.26 to 0.50)
60	0.64 (0.54 to 0.72)	0.51 (0.43 to 0.69)	1.16 (0.55 to 1.52)	0.47 (0.40 to 0.87)	0.33 (0.31 to 0.38)	0.35 (0.27 to 0.44)

*RMS error was calculated on the basis of Eq. 10. The second order fit (*v*_*mdl*,2_) presents the lowest median (IQR) for both *v*_*est*_ and *v*_*R*_.*

**FIGURE 7:**
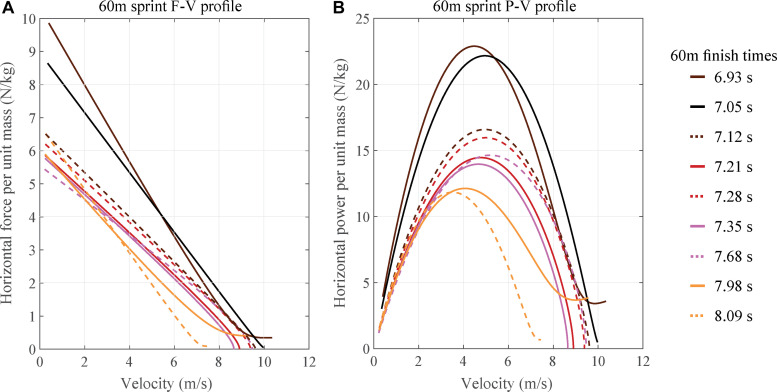
**(A)** Horizontal force (per unit mass) – velocity profile for the respective best 60 m performance of nine athletes. **(B)** Power (per unit mass) – velocity profile, based on second order exponential fit, for the respective best 60 m performance of nine athletes.

Force-velocity (F-V) and power-velocity (P-V) obtained from the second-order (order 2) exponential are shown in Figure 7, respectively. These profiles were created from the best trial of the nine selected athletes for the 60 m sprint and sorted from the lowest to the highest finish times.

## Discussion

### Validity of the Proposed Method

The proposed sensor-fusion algorithm can compute an accurate velocity profile with respect to the radar; it can compensate for and improve upon the accuracy of the individual IMU and GNSS velocities, as seen in Figure 3B. When *v*_*GNSS*_ is relatively accurate, the algorithm output (*v*_*est*_) closely resembles the *v*_*GNSS*_ profile (Figure 3C). This is underlined by the percentage error for the velocity (Figure 4A); the median RMS error values for the *v*_*est*_ are only slightly lower than those for *v*_*GNSS*_, whereas the standard deviation is considerably less. Thus, the velocity estimation algorithm based on GNSS and IMU fusion is robust in terms of accuracy and precision, despite the inaccuracies in the GNSS velocity. None of the previous works on estimation of sprint mechanics (Samozino et al., 2016; Stanton et al., 2016; Gurchiek et al., 2018; Setuain et al., 2018) conducted a validation of the instantaneous velocity or the overall profile with respect to a speed radar. Stanton et al. (2016) validated the mean velocity over an entire sprint, while (Gurchiek et al., 2018) validated the mean velocity over 10 m intervals. This method is the first one to provide validated instantaneous analysis of the sprint velocity profile over multiple distances, and thus it is not possible to compare our results with the state-of-the-art.

The median error for sprint duration (*T*_*est*_) increased from 0.1 to −6.3% for 30 to 60 m distances, respectively, clearly showing an overestimation. This is a result of the minor underestimation of velocity caused by the residual drift in the IMU strapdown integration and the inaccuracies of the GNSS velocity. While the work by Setuain et al. (2018) used photocells for drift estimation, only the research from Stanton et al. (2016) considered a validation with respect to the photocell data. The mean error reported in the latter case (2.6%) for 10 m sprint was higher than the median (IQR) error presented here i.e., 0.1 (−1.7 to 1.9) (Table 1) for a 30 m sprint. Furthermore, it was validated solely for 10 m sprints and the algorithm was focused only on the calculation of the mean velocity. The median error and IQR for estimated sprint duration (*T*_*est*_) is higher than the one obtained from the speed radar (*T*_*R*_), except for 30 m sprint where the median error is lower (Table 1). Thus, the algorithm is less robust than the radar. This might be the result of the assumption of purely sagittal plane motion, which can be violated to different degrees by the different magnitude of mediolateral motion resulting from the varied running techniques of the sprinters.

Comparison of the estimated maximum velocity to that from the radar (Figure 4C) showed a bias of −0.12 m/s, which is in agreement with the slight underestimation of velocity discussed in the preceding paragraph and lower than the 0.20 m/s value reported in Gurchiek et al. (2018). Despite this bias, the estimated maximum velocity showed a “strong” agreement with the measured one, indicated by the magnitude of the Lin’s concordance correlation coefficient (ccc) being 0.76 (*p* < 0.05). In comparison, (Setuain et al., 2018) compared the estimated maximum velocity with the measured one, obtaining a ccc value of 0.81 (*p* < 0.05). However, the maximum velocity in this work was estimated indirectly through a linear force-velocity relationship based on the first order exponential fit model (Eq. 4) for both, the IMU and the reference force plate data. For the *v*_*max*_, the limits of agreement (L.O.A.) for the Bland–Altman plot ranged from −1.20 to 0.89 m/s, this range being smaller than one (−1.25 to 1.64 m/s) presented in Gurchiek et al. (2018). L.O.A for the *v*_0_ parameter varied from −1.01 to 0.67 m/s, which is similar in extent to one (−0.7 to 1.3 m/s) showed in Samozino et al. (2016). The *f*_0_ and *p*_*max*_ magnitudes were computed in terms of per unit mass and hence the L.O.A cannot be directly compared to the ones from (Samozino et al., 2016).

### Exponential Fitting and Athlete Profiles

Use of a first order exponential fit (Samozino et al., 2016; Setuain et al., 2018) is the dominant method of estimating the sprint velocity profile and subsequently the force (*F*)-power (*P*)-velocity (*V*) relationships. In this work, we compared the accuracy of this first order exponential (Eq. 1) and a second order exponential (Eq. 6) in approximating the velocity profile produced by our algorithms and by the reference radar system. Figure 6A showed the second order fit to better approximate the velocity profile, while the first order fits led to an underestimation of the velocity. For all sprint distances, the median RMS error for second order exponential was consistently less than that for the first order exponentials; this was true for both fits based on *v*_*R*_ or *v*_*est*_. The error values are different across athletes and different sprint distances, emphasizing the idea that the velocity profile does not necessarily present first order exponential behavior. While the first order fit is suitable to represent a maximal effort during sprint competitions (Samozino et al., 2016), the athletes may not necessarily undertake a maximal effort during training sessions. Thus, a second order exponential can offer a truer representation of the sprinter’s velocity profile across different contexts. However, estimating the three variables (*a*,τ_1_,τ_2_) in Eq. 6 is an optimization problem, leading to a higher computational cost than solving the Eq. 1 for a single variable τ. In case of real-time processing, this added complexity could be detrimental.

Use of a first order exponential leads to linear F-V and parabolic P-V profiles, which have been investigated previously (Morin and Samozino, 2016) for their potential to predict risk of injury and to plan training goals. The second-order exponential leads to more accurate albeit non-linear F-V and non-parabolic P-V profiles, as seen in Figure 7. As expected, the area under the curve for both profiles is higher for athletes with lower finish times and vice-versa. For the top two athletes (6.93 and 7.05 s), the F-V profile (Figure 6B) shows an interesting contrast, one (6.93 s) of them starts with a higher acceleration, has a stronger reduction in the same, and yet the athlete continues to accelerate throughout the 60 m. Whereas the second (7.05 s) athlete starts with a lower acceleration but has a slower reduction in its magnitude. Such differences, when observed over multiple trials, can help in identifying the strengths and the areas of improvement for athletes. Whether the increased accuracy resulting from the second order exponential improves the analysis of athletes is a potentially important practical research question for sports scientists.

### Limitations and Future Work

The two main limitation of the proposed algorithm arise primarily out of the gradient descent (Madgwick et al., 2011) procedure used for converting the IMU acceleration from the sensor frame to the global frame. First, this procedure necessitates the use of magnetometer for reliable estimation of the acceleration in the lateral direction. We assume that the motion occurs purely in the sagittal plane, thus negating the necessity of using lateral acceleration and simplifying the process model in the Kalman filter to a one-dimensional linear model. This assumption holds because of the approximate straight-line motion of the sprinter; it also forms the basis of radar-based velocity measurement. Thus, the proposed algorithm is valid for straight-line sprints and not for curve sprinting or sprints with direction changes. Second, the gradient descent uses a static period to determine the orientation with respect to gravity and thus the algorithm is sensitive to the selected starting point of the sprint. Thus, absence of a static period before the start of the sprint can lead to unreliable conversion of the acceleration to the global frame. To ensure the availability of this static period, we visualize the raw GNSS velocity plot and manually select the starting point for the segmentation of the sprint data. However, an automated segmentation procedure, possibly based on the GNSS velocity, can allow for a more robust and repeatable segmentation, and subsequently enable a more accurate estimation of sprint velocity. Automated segmentation can also simplify the analysis when a battery of tests, such as the agility *T*-test (Pauole et al., 2000), the sprint test, and the bleep test (Bangsbo and Krustrup, 2001), are performed together. This is typically the case for pre-season testing in team sports such as soccer, rugby, hockey, etc. The limited sample size of this study constitutes the last limitation. However, this study is aimed strictly toward the technical validation of the proposed algorithm and we attempted to overcome this limitation by conducting multiple trials per participant. While this study was mainly focused on the algorithm development and validation, there is definitely a potential for a follow-up study with different groups of sprinters of varied skills to test the discriminatory power of the results from the algorithm.

## Conclusion

The goal of our study was accurate estimation of the sprint velocity profile using a back-worn GNSS-IMU sensor and its validation with the reference system i.e., a Doppler speed radar. To overcome the individual limitations of the GNSS and IMU sensors, we utilized a sensor-fusion approach based on Kalman filter to fuse the GNSS velocity and the IMU acceleration signals. We achieved velocity profile estimation with a median error ranging from 6.14 to 8.11% respect to the radar speed profile, for sprint distances varying from 30 to 60 m. Additionally, we showed an improved approximation of the velocity profile using a second order exponential model, thus raising doubts over the dominant approach of using a first order exponential model. Further studies should investigate the advantage of utilizing second order exponential model in athlete training and monitoring. To extend this work in future, we may automate the segmentation procedure and use the IMU signals to analyse the gait temporal parameters. By pursuing this path, we hope to augment the potential of sprint test used in training to assess injury risk of athlete and improve their performance.

## Data Availability Statement

The raw data supporting the conclusions of this article will be made available by the authors to qualified researcher, without undue reservation.

## Ethics Statement

The studies involving human participants were reviewed and approved by EPFL Human Research Ethics Committee (HREC 039-2018). The patients/participants provided their written informed consent to participate in this study.

## Author Contributions

SA, FM, FD, and KA conceptualized the study design, while SA and FM conducted the data collection. SA designed and implemented the algorithms with KA and FD guiding the study. SA, FM, FD, VG, and KA contributed to the analysis and interpretation of the data. SA drafted the manuscript, with inputs from FM, FD, VG, and KA on its outline and structure. All authors revised it critically, approved the final version, and agreed to be accountable for all aspects of this work.

## Conflict of Interest

FD was employed by company Gait Up S.A.

The remaining authors declare that the research was conducted in the absence of any commercial or financial relationships that could be construed as a potential conflict of interest.

## Appendix

### Sensitivity Analysis

We conducted a sensitivity analysis to examine the change in the percentage RMS error with a corresponding change in the threshold (section “Velocity and Duration Estimation Algorithm”) used to detect the start of the sprint. We tested the algorithm for a range of thresholds around the chosen value of 0.3 m/s (Figure 7), increasing in steps of 0.05 m/s, from 0.2 to 0.4 m/s. For the 30 m sprint, the algorithm showed around 1% change in RMS error at thresholds lower than the chosen one but no almost no change for threshold higher than 0.3 m/s. For 40 and 60 m sprints, the RMS error resulting from the algorithm presented almost negligible sensitivity to the threshold.

## Abbreviations

*a*_*mdl*_: acceleration from model
*v*_*mdl_max, 1*_: first order model based on maximum velocity
*v*_*mdl_end, 1*_: first order model based on speed end
*a*_*GFx*_: forward acceleration in global frame
*v*_*est*_: IMU-GNSS fusion estimated speed
*v*_max_: maximum velocity
*v*_*R*_(*t*): radar speed
*v*_*mdl, 2*_: second order model
*v*_*end*_: velocity at the endpoint of sprint
*v*_*GNSS*_: velocity measured by GNSS
*v*_*mdl*_: velocity of the exponential model.
